# Editorial: Latest findings on *Leishmania* parasites for better vaccine design and drug development

**DOI:** 10.3389/fmicb.2025.1554339

**Published:** 2025-01-30

**Authors:** Negar Seyed, Tahereh Taheri, Farhat Afrin, Hamidreza Majidiani

**Affiliations:** ^1^Department of Immunotherapy and Leishmania Vaccine Research, Pasteur Institute of Iran, Tehran, Iran; ^2^Centre for Interdisciplinary Sciences Faculty, JIS Institute of Advanced Studies & Research, West Bengal, India; ^3^Healthy Aging Research Centre, Neyshabur University of Medical Sciences, Neyshabur, Iran; ^4^Department of Basic Medical Sciences, Faculty of Medicine, Neyshabur University of Medical Sciences, Neyshabur, Iran

**Keywords:** *Leishmania*, concomitant immunity, vaccine, drug repurposing, drug non-responsiveness, infectious models

*Leishmania* is a well-known unicellular parasite as the causative agent of a debilitating vector-borne disease with diverse manifestations from fatal visceral (VL) and mucocutaneous (MCL) forms to self-healing cutaneous forms (CL). There is an urgent need for protective vaccines and also drug candidates due to the changes in the map of endemicity and world-wide spread of the disease (Paz, 2024). However, failure in the deep understanding of real parasite-host-vector interaction has hampered the development of protective vaccines or effective treatments. Seyed et al. have discussed some of the underlying reasons of vaccination failure and the correlates of protection already identified in mouse models and the vaccine formulations that better meet these protection criteria namely Live attenuated or non-pathogenic *Leishmania* species and DNA vaccines. The application of new technologies such as CRISPR-Cas9 (Sharma et al., 2021) and new generation of antibiotic-free plasmids (Alonso et al., 2023) are now available to contribute in solving the inbuilt drawbacks associated with these vaccine platforms. Basically, protective vaccines against *Leishmania* or other relevant macrophage-resident parasites such as *Trypanosoma cruzi* benefit from “concomitant immunity” which means “persistent, low-level infection” (Peters and Sacks, 2009; Peters et al., 2014; Seyed and Rafati, 2021). Cai et al. have demonstrated the effectiveness of an experimental live vaccine against chagas disease formulated on a recombinant avirulent *Leishmania major* (*dhfr*−*ts*^−^) which expresses the *Trypanosoma cruzi* antigen. The outcome of the study warrants further investigation of the live-attenuated *Leishmania* as vaccine to meet the “concomitant immunity” for both leishmaniasis and chagas, two globally important infections.

For the moment, while human vaccines lag behind, chemotherapy still plays the most important role in disease control. However, rising resistance to the current therapeutics, urges new chemicals to be replaced. Despite significant breakthroughs in high throughput drug discovery, there is an urgent need for identification of promising novel anti-*leishmania* compounds. Almeida Machado et al. have advantaged drug repurposing which involves identifying new therapeutic uses for existing drugs that are already approved for other indications (Kulkarni et al., 2023). This group has presented for the first time the *in vitro* effectiveness of subtilisin inhibitor (PF-429242), an anti-viral drug, against *Leishmania* infantum parasite. PF-429242 suppresses an important protease and induces several cellular alterations such as mitochondrial damage, oxidative stress and autophagy in addition to modulating the host immune response. Recently, synthetic biology has come into focus to enhance the development of vaccines against leishmaniasis, *Leishmania* diagnostic tools and novel therapeutics including immunetherapeutics. Synthetic biology is a multidisciplinary field of science that focuses on living systems and organisms, and it applies engineering principles to develop new biological parts, devices, and systems or to redesign existing systems found in nature (Hanczyc, 2020). Khandibharad and Singh, explain some of the applications of synthetic biology in combat against leishmaniasis which have resulted in promising pre-clinical achievements like synthetic circuits and synthekines. Besides intensive investigation on new drug discovery, Bamorovat et al. have introduced the risk factors that predispose to meglumine antimoniate unresponsiveness in *Leishmania tropica* derived CL. Accordingly, the treatment outcome is not only dependent on the resistance genes of the parasite (Nery et al., 2024) but also on the demographical, clinical and environmental factors, host immune response, adherence to treatment by patients, genetic background and even RNA virus infection in parasites. Therefore, for any treatment (existing or upcoming), regular field monitoring for resistance factors to prevent unresponsiveness is crucial (Wijnant et al., 2022).

The last but not the least is the critical role that experimental models play in evaluating the efficacies of newly designed and developed vaccines or treatments. For some *Leishmania* species, a preclinical model with human leishmaniasis characteristics is still missing, for example there are no reported standardized models specific to mucocutaneous leishmaniasis (Suckow et al., 2024). Species of *Viannia* subgenus are poorly infective in mice and are generally asymptomatic or induce a mild disease that resolves within few weeks with almost elimination of the parasite, a condition that weakly resembles a human disease (de Oliveira and Brodskyn, 2012). Munoz-Durango et al. have explained the development of a CL model that uses a mouse adapted *Leishmania panamensis* isolate to reproducibly induce a dermal disease very similar to human CL in BALB/c mice. They have confirmed that this model suits to evaluating the pharmacological and vaccine interventions regarding *Viannia panamensis*.

To conclude, our current understanding of antileishmanial correlates of protection is not complete. This necessitates first the full monitoring of the emerging unresponsiveness to current therapies to maintain the competence of existing drugs in endemic areas. Second, we must rush into novel drug development to substitute the unaffordable high-risk therapies already in use especially in visceral leishmaniasis endemic areas. Advanced technologies such as single-cell analysis and controlled human infection models (CHIM) will hopefully make vaccine dream come true in near future.
